# In-Wheel Motor Fault Diagnosis Method Based on Two-Stream 2DCNNs with DCBA Module

**DOI:** 10.3390/s25154617

**Published:** 2025-07-25

**Authors:** Junwei Zhu, Xupeng Ouyang, Zongkang Jiang, Yanlong Xu, Hongtao Xue, Huiyu Yue, Huayuan Feng

**Affiliations:** School of Automotive and Traffic Engineering, Jiangsu University, Zhenjiang 212013, China; zhujw@stmail.ujs.edu.cn (J.Z.); oyxp@stmail.ujs.edu.cn (X.O.); jzk@stmail.ujs.edu.cn (Z.J.); xuyanlong@stmail.ujs.edu.cn (Y.X.); brendonyueh@163.com (H.Y.); fhy@ujs.edu.cn (H.F.)

**Keywords:** in-wheel motor, fault diagnosis, two-stream 2DCNNs, depthwise convolution block attention

## Abstract

To address the challenge of fault diagnosis for in-wheel motors in four-wheel independent driving systems under variable driving conditions and harsh environments, this paper proposes a novel method based on two-stream 2DCNNs (two-dimensional convolutional neural networks) with a DCBA (depthwise convolution block attention) module. The main contributions are twofold: (1) A DCBA module is introduced to extract multi-scale features—including prominent, local, and average information—from grayscale images reconstructed from vibration signals across different domains; and (2) a two-stream network architecture is designed to learn complementary feature representations from time-domain and time–frequency-domain signals, which are fused through fully connected layers to improve diagnostic accuracy. Experimental results demonstrate that the proposed method achieves high recognition accuracy under various working speeds, loads, and road surfaces. Comparative studies with SENet, ECANet, CBAM, and single-stream 2DCNN models confirm its superior performance and robustness. The integration of DCBA with dual-domain feature learning effectively enhances fault feature extraction under complex operating conditions.

## 1. Introduction

It is generally known that electric vehicles have become a key means to solve traffic pollution and sustainable use of energy and have been a research focus for automobile manufacturers and institutes. Thereinto, the drive system is an important component. Different drive system layouts of electric vehicles have recently emerged to improve efficiency, simplify structure, and save space [1,2]. The four-wheel independent drive system is a new mode that can eliminate some transmission parts and improve the utilization of space and power transmission efficiency, especially for in-wheel motor drive systems, where each in-wheel motor is directly connected to the drive wheel, allowing independent control for high efficiency, fast response, and full-time wire control. As such, it has become a research hotspot in the development of new energy usage in the automobile industry [3,4,5]. However, the changeable driving conditions and harsh operating environment not only aggravate the impact of suspension and road surface on the stator and rotor of an in-wheel motor, but also deepen the contradiction between the heat dissipation and seal, which can easily induce mechanical failure and cause vehicle safety accidents in serious cases [6,7]. This aligns with the two-stage degradation modeling approach and provides insights into identifying key degradation angles for accurate fault diagnosis [8]. Therefore, it is vital to establish an online fault diagnosis system for in-wheel motors to monitor the running state and identify common faults.

There are a lot of studies on the fault diagnosis of complex electromechanical equipment such as motors, generators, and transformers, but there are very few methods for diagnosing potential in-wheel motor faults in changeable driving conditions and harsh operating environments. Though many research results can be used as references, they have poor adaptability and their diagnostic accuracy is not satisfactory. It is well known that a rolling bearing is an important part in rotating machines, and that bearing defects are also the most common mechanical fault; three bearing defects are often taken as the identification objects to present many fault diagnosis methods. For example, time-domain analysis [9,10], frequency-domain analysis [11,12,13], and time–frequency-domain analysis [14,15] are based on signal processing and feature extraction for capturing the vibration characteristics generated by bearing faults. The grayscale images reconstructed from vibration signals in different domains are used to extract multiple features such as the most prominent features, more local features, and average information, which is inspired by the adaptive multi-sensor and multi-feature fusion strategy [16]. The bidirectional weighted enhanced envelope spectrum analysis method is proposed to enhance the resonance frequency band and the fault characteristic frequency of bearing signals [17]. The dual-kurtogram algorithm and multivariate statistical process control are used to establish a method for condition monitoring and compound fault diagnosis of rolling bearings, with the core idea focusing on the sub-band extraction capability of the dual-kurtogram algorithm [18]. The sparse Bayesian learning (SBL)-based method is proposed for the compound bearing fault diagnosis; specifically, a novel categorical probabilistic model is first introduced to effectively identify the actual fault components by constraining the solution space within a truncated feasible domain. Subsequently, a more generalized SBL framework is developed to reconstruct the compound sparse impulse signal under this probabilistic model [19]. An adaptive two-stage degradation framework based on Gaussian process regression is proposed to adequately describe complex degradation process and provide reliable predictions of remaining useful life; this framework dynamically selects the optimal degradation model according to the observed characteristics of the degradation data, thereby enhancing both prediction accuracy and adaptability [20]. Variational mode decomposition and wavelet thresholding are combined to denoise vibration signal, time–frequency continuous wavelet transform is used to generate scalogram images, and deep learning is employed to classify and diagnose bearing faults [21]. Complete ensemble empirical mode decomposition is improved with adapted noise to decompose the vibration signals into several intrinsic modal functions; permutation entropy is introduced as the fault feature of rolling bearings [22]. A convolutional neural network (CNN) steered with wavelet synchrosqueezing transform (WSST)-based scalograms is proposed to successfully diagnose the various bearing faults in an electric two-wheeler [23]. Spectrum alignment and deep transfer convolutional neural networks are used to present a novel fault diagnosis method for solving the problem of category imbalance and various speeds concurrently [24]. However, the above methods are easily affected by prior knowledge and can cause the loss of some useful information, leading to unstable performance, generalization, and robustness.

Certainly, a small number of research papers on the feature extraction and fault diagnosis of typical in-wheel motors’ faults have also emerged. For example, non-negative matrix factorization is refined to propose an adaptive signal reconstruction strategy for compound fault detection of in-wheel motors’ bearings by adopting the Itakura–Saito distance and the sparse constraint [25]. An original feature extraction method based on optimized singular spectrum decomposition and enhanced adjusted multipoint optimal minimum entropy deconvolution is proposed to realize the fault detection of in-wheel motors’ bearings [26]. Support vector data description (SVDD) is improved using the Weibull kernel function to present a novel diagnosis method for monitoring each in-wheel motor fault [27]. These methods provide important references for extracting the fault features of in-wheel motors. When applied in engineering practice, there are still some technical problems that need to be solved urgently.

With the development of artificial intelligence, many network models of fault diagnosis have been built to get rid of the dependence on expert knowledge, especially deep learning theory [28,29,30]. For example, the deep diversity maximization-based adversarial transfer diagnosis approach for rotating machinery is presented to solve trivial solutions of the existing transfer diagnosis methods based on entropy minimization; its core idea lies in employing a deep convolutional neural network as a feature encoder to extract discriminative characteristics of vibration signals under varying operating conditions [31]. Moreover, a novel transfer learning network based on CNNs is introduced to tackle the challenge of limited fault data availability for rolling bearings in constrained industrial environments [32]. The SBL framework is presented to exploit the group-sparsity and additional periodicity behavior of fault impulses, and then the hyperparameters are automatically tuned without any prior knowledge; a maximum posteriori estimator can be obtained in the sense of Bayesian optimality to yield higher accuracy [33]. A novel fault diagnosis method based on an improved auxiliary classification generative adversarial network is proposed to address the issue of insufficient training samples in the field of bearing fault diagnosis [34]. Gramian angular summation fields are employed to transform multi-sensor signals into 2D feature maps for mitigating feature distortion and enhancing noise elimination for early detection, and deep belief network and minimum unscented Kalman filters are utilized to construct a robust deep learning framework capable of analyzing the translated 2D feature maps for effective diagnosis of early bearing faults [35]. Actively imaginative data augmentation is proposed to address the challenge of intelligent fault diagnosis under large-speed-fluctuation conditions, which involves two adversarial training stages: knowledge learning and sample imagining [36]. A generative adversarial network model is proposed to generate sufficient synthetic samples for the minority faulty conditions so as to rebalance the datasets [37]. A motor fault detection scheme based on a one-class tensor hyperdisk model is proposed to address the issues of low recall for normal samples, which manifest as a high false alarm rate and reduced overall detection accuracy [38]. The above methods share the trait of the input information being relatively singular, such as types of monitoring signal, the status information from a sensor, or the fault features identified by analyzing in a domain. However, the diagnostic accuracies of these methods struggle to meet practical engineering requirements when applied to complex scenarios characterized by varying operating conditions, particularly within the context of electric vehicles.

In recent years, two-dimensional CNNs (2DCNNs) have been increasingly applied in the field of fault diagnosis, demonstrating their significant potential in intelligent fault recognition and classification tasks [39,40]. Leveraging automatic feature extraction and powerful pattern recognition capabilities, 2DCNNs enable more accurate and efficient equipment condition monitoring and fault detection. For example, 2DCNNs’ automatic feature-extraction capability and the robust discrimination performance of random forest classifiers are utilized to propose a fault-diagnosis strategy with good noise immunity for solving the fault detection and diagnosis of offshore wind-turbine high-speed bearings under a complex environment and structural loads [41]. A network structure algorithm based on a combination of a 2DCNN and hybrid kernel fuzzy support vector machine (HK-FSVM) is proposed to address the limitations of traditional fault diagnosis methods, particularly their poor noise robustness and inadequate extraction of fault information from vibration signals [42]. These methods usually focus on extracting the key features from image information in a single domain, but they ignore information from multiple sensors or multiple domains.

Furthermore, attention mechanisms (AMs) have been increasingly integrated into 2DCNN architectures, demonstrating notable advantages in feature extraction, computational efficiency, model generalization, and robustness. Especially when dealing with complex image data [43], fault diagnosis [44] and anomaly detection [45], AM can help the model focus on the important areas in the image, thereby improving the diagnostic accuracy and performance. Common AMs include self-attention, additive attention, dot-product attention, multi-head attention, channel attention, spatial attention, etc. Different types of AMs apply varying importance weights to input data, allowing the model to emphasize critical features more effectively. In the application of 2DCNNs, AMs usually help the model capture the key information or important areas in the image, thereby improving the performance of the model. Facing different computing resources and task requirements, some new AMs such as squeeze-and-excitation networks (SENets) [46], efficient channel attention networks (ECANets) [47], an convolutional block attention modules (CBAMs) [48] have emerged, which enhance feature learning through an adaptive approach, thereby improving the performance of the model in tasks such as object detection and fault classification. However, when directly applied to the recognition of multiple in-wheel motor faults, the outcomes of these AMs have proven unsatisfactory.

Therefore, this paper proposes a novel diagnosis method based on two-stream 2DCNNs with a DCBA (depthwise convolution block attention) module for accurately recognizing the faults of each in-wheel motor in a four-wheel independent driving system under a real scenario of changeable driving conditions and harsh operating environment, the creativity of which mainly manifests in two aspects. One of the key contributions is the proposal of a novel attention mechanism, termed DCBA, which is designed to emphasize discriminative fault features from multi-sensor vibration signals in both the time domain and the time–frequency domain. Another is to present a new network structure of two-stream 2DCNNs with a DCBA module; each stream’s 2DCNN is responsible for extracting the excellent fault features from a domain, the fully connected layer is used to fuse the extraction features, and then the high-precision identification of in-wheel motors’ faults is realized. The rest of this paper is organized as follows. Section 2 gives the theoretical background. Section 3 provides the proposed network structure of two-stream 2DCNNs with a DCBA module for in-wheel motor fault diagnosis. Section 4 investigates the effectiveness of the proposed fault diagnosis method. Finally, conclusions are drawn in Section 5.

## 2. Theoretical Background

To better outline the fault diagnosis method proposed in this paper, the basic theories such as 2DCNN and attention mechanisms (AMs) are reviewed, especially when multi-channel maps are used as input data.

### 2.1. 2DCNN

2DCNNs are a type of deep learning model that have fewer parameters and directly use raw images as input data, making them widely applicable in image processing tasks. In general, 2DCNNs include convolutional layers, pooling layers, and fully connected layers [39]. In convolutional layers, a series of kernels are employed to capture the local features in the image. The convolution operation generates a feature map by sliding a kernel across the image, performing element-wise multiplication and summation between the pixel values of each local region and the corresponding kernel weights. This method of local connection and weight sharing can reduce the number of model parameters and improve the computational efficiency. At the same time, the convolution operation helps preserve the spatial structure of the input image, which is essential for effective feature representation. Let the *lth* layer be a convolutional layer; the convolution process can be expressed as follows:

(1) $$Y_{c}^{l+1}(i,j)=f(\sum_{m}\sum_{n}\left(X_{c}^{l}((i-1)s+m+1,(j-1)s+n)\cdot K_{c}^{l}(m,n)+b_{c}^{l}\right))$$

where $X_{c}^{l}$ is the feature map in the *c*th channel and the *l*th layer, and $Y_{c}^{l+1}$ is the convolutional output in the *l*th layer, and is denoted as the input in the (*l* + 1)th layer. *c* = 1, 2, 3, …, *C*, *C* is the number of channels. $K_{c}^{l}$ and $b_{c}^{l}$ are the kernel and bias in the *c*th channel and the *l*th layer. *s* is the stride, *i* and *j* are the row and column indexes of $y_{c}^{l}$, and *m* and *n* are the row and column indexes of $K_{c}^{l}$, respectively. Let *H* and *W* be the row and column sizes of $X_{c}^{l}$, and *k_H_* and *k_W_* be the row and column sizes of $K_{c}^{l}$; then, *m* = 1, 2, …, *k_H_*, *n* = 1, 2, …, *k_W_*, and the row and column sizes of $Y_{c}^{l+1}$ can be confirmed as $\left\lfloor (H+2p-k_{H})/s\right\rfloor +1$ and $\left\lfloor (W+2p-k_{W})/s\right\rfloor +1$. Moreover, $f\left(\cdot \right)$ represents the activation function, such as sigmoid, tanh, softmax, swish, and rectified linear unit (ReLU). In this paper, gradient characteristics and computational efficiency are considered, and ReLU is selected to enhance the divisibility of the extracted features.

A pooling layer usually follows the convolutional layer, as it plays a critical role in compressing the feature map. Currently, various pooling methods have been developed, such as max-pooling, average pooling, logarithmic pooling, and weighted pooling. To extract dominant features, reduce the number of network parameters, and enhance computational efficiency, max-pooling is employed in the pooling operation of the proposed method, which is formulated as follows:

(2) $$P_{c}^{l+1}(i,j)=\underset{m,n=1,2,\ldots ,\Omega }{\max}\left\{Y_{c}^{l}((i-1)s+m,\;(j-1)s+n)\right\}$$

where $P_{c}^{l+1}$ is the pooling result in the *c*th channel and the *l*th layer, Ω is the width of the pooling window, and $p_{r}^{l+1}$ is the result of the pooling layer.

Finally, the fully connected layer serves the purpose of fully connecting all neurons in the network. Normally, the fully connected layer is also linked to the output layer and performs a classifier. Certainly, various functions can be utilized for classification and recognition tasks. In this paper, the softmax function is selected as the classifier, and its output can be mathematically defined as follows:

(3) z=σ(ωP+b)

where *z* is the output vector in the fully connected layer, *P* is the input tensor in the fully connected layer that is composed of $P_{c}^{l+1}$ in every channel, $P=\left\{P_{1}^{l+1},\;P_{2}^{l+1},\;\ldots ,P_{c}^{l+1},\;\ldots \right\}$. *σ* is the softmax function, and *ω* and *b* are the weight matrix and bias vector, respectively, which are constantly optimized by a backpropagation algorithm to minimize the loss function.

### 2.2. Attention Mechanism (AM)

AMs are novel techniques that have achieved significant success in deep learning, particularly in natural language processing and computer vision [44]. In recent years, AMs have been widely used in CNNs to improve performance in image processing tasks by enhancing the ability to focus on important features in the input data. There are many typical applications such as SENet, ECANet, and CBAM. Therefore, SENet is an important milestone in deep learning that marks the formal introduction of AM applications in model design [46]. The operation can be generalized as follows:

(4) $$\tilde{X_{c}}=\mathrm{Sigmoid}(W_{2}\cdot \mathrm{ReLU}(W_{1}\cdot \mathrm{GAP}(X_{c})))\cdot X_{c}$$

where *X_c_* is the feature map in the *c*th channel, $\tilde{X_{c}}$ is the output feature map, GAP(·) is the function of global average pooling (GAP), ReLU(·) and sigmoid(·) are the activation functions of ReLU and sigmoid, and *W*_1_ and *W*_2_ are the corresponding weight matrixes in the fully connected layer, respectively.

ECANet proposes a more efficient AM that replaces the fully connected layer with a one-dimensional convolution [47], thereby reducing the number of parameters and computation. The calculation can be summarized as follows:

(5) $$\tilde{X_{c}}=\mathrm{Sigmoid}(\mathrm{Conv}1D(\mathrm{GAP}(X_{c}),\;k))\cdot X_{c}$$

where Conv1D(·) is a one-dimensional convolution, and *k* is the size of the convolution kernel.

CBAM improves the model’s ability to capture important features by combining channel attention and spatial AMs. It is structurally simple and easily integrable, and thus can be seamlessly inserted into existing CNN architectures as an add-on module [48]. CBAM is mainly composed of two sub-modules such as channel attention modules and spatial attention modules, which operate on the input feature map in turn. The integral calculation process can be described as follows:

(6) $$X_{c}^{\prime }=\mathrm{Sigmoid}(W_{2}\cdot \mathrm{ReLU}(W_{1}\cdot \mathrm{GAP}(X_{c}))+W_{2}\cdot \mathrm{ReLU}(W_{1}\cdot \mathrm{GMP}(X_{c})))\cdot X_{c}$$

(7) $$\tilde{X_{c}}=\mathrm{Sigmoid}(\mathrm{Conv}([\mathrm{GAP}(X_{c}^{\prime }),\;\mathrm{GMP}(X_{c}^{\prime })];\;k\times k,\;s))\cdot X_{c}^{\prime }$$

where GMP(·) is the function of global maximum pooling (GMP), [·,·] denotes the concatenation operation of two elements, Conv(·) is a convolution operation, *k* is the size of the convolution kernel, and *s* is the stride.

These AMs can be integrated into different parts of a CNN, including the channel and spatial dimensions, to meet specific task requirements. The primary goal is to enhance the CNN’s performance by focusing on important features in the input data, while keeping computational overhead minimal through the use of lightweight modules. Therefore, the application of AMs in CNNs has become an important means to improve model performance.

However, SENet and ECANet rely solely on GAP to extract global average features, potentially overlooking the most salient ones. CBAM incorporates both global average and max features, yet lacks the flexibility to adaptively focus on different regions of the feature map based on task-specific requirements.

## 3. The Proposed Approaches

The fault diagnosis method of each in-wheel motor in the four-wheel independent driving system proposed in this study mainly includes two methods. Firstly, a novel AM called depthwise convolution block attention (DCBA) is proposed to enhance the focus on fault-related features. Secondly, a fresh network structure of two-stream 2DCNNs with a DCBA module is built to extract the fault features in the time domain and time–frequency domain. More importantly, three DCBA modules are inserted in different places according to the model architecture and the weighting task of effective fault features.

### 3.1. Depthwise Convolution Block Attention (DCBA)

**Figure 1:**
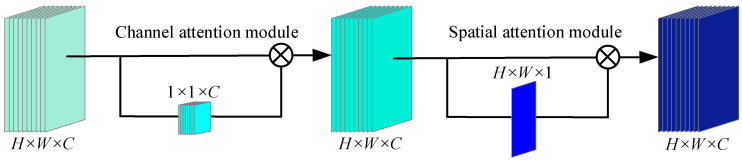
The DCBA framework proposed in this paper.

To efficiently extract the fault features in changeable driving conditions and a harsh operating environment for in-wheel motor fault diagnosis, a DCBA module is proposed. Figure 1 shows the DCBA’s framework, which includes two modules that are channel attention and spatial attention modules in turn. The channel attention module mainly learns a 1 × 1 × *C* channel attention map to measure the important features of each channel. The spatial attention module mainly infers a *H* × *W* × 1 spatial attention map to emphasize the distinctive features in each location.

**Figure 2:**
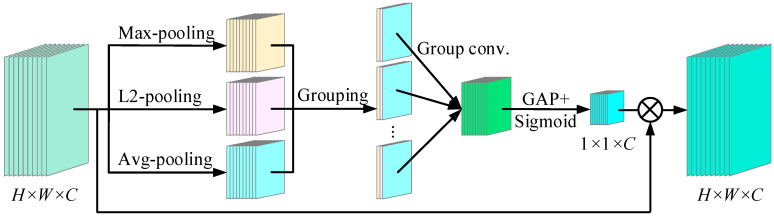
Depthwise convolution channel attention module.

In channel attention, many existing methods simply employ GAP or GMP to refine channel features. In this way, more local features are usually ignored, which is not conducive to fault diagnosis. Therefore, a depthwise convolution channel attention module is proposed, as illustrated in Figure 2. The overall operation consists of four stages: pooling, grouping, group convolution, and GAP. In the first stage, max-pooling, L2-pooling and avg-pooling operations are employed to learn the features of each channel from different dimensions. In particular, L2-pooling generates the output map by calculating the Euclidean norm for all elements in the pooling window. The advantage is that more local feature information can be retained, which is suitable for scenarios where details need to be modeled. Then L2-pooling is selected to extract the fault feature. The formula describing the working principle of L2-pooling is as follows:

(8) $$L2\_p(X)=\sqrt{\frac{1}{k^{2}}\sum_{m=0}^{k-1}\sum_{n=0}^{k-1}{\left(X(m,\;n)\right)}^{2}}$$

where X(*m*, *n*) is the element at the *m*th row and *n*th column in the feature map *X*.

In the second stage, the three pooling results of each channel within the same group are aggregated, and there are a total of C groups. In the third stage, a standard group convolution operation is performed in each group, and the convolutional outputs are arranged in channel order. In the fourth stage, GAP and a sigmoid function are applied to generate a 1 × 1 × C channel attention map. The depthwise convolution channel attention module is computed as follows:

(9) $${X^{\prime }}_{c}=\mathrm{Sigmoid}(\mathrm{GAP}(\mathrm{Conv}([\max\_p(X_{c}),\;L2\_p(X_{c}),\mathrm{avg}\_p(X_{c});\;k\times k,\;s];\;k^{\prime }\times k^{\prime }\times 3,\;s^{\prime })))\cdot X_{c}$$

where max_p(·), L2_p(·) and avg_p(·) are the functions of max-pooling, L2-pooling, and avg-pooling, respectively. *k* × *k* and *k*’ × *k*’ × 3 are the sizes of the pooling window and convolution kernel, and *s* and *s’* are the corresponding strides, respectively.

**Figure 3:**
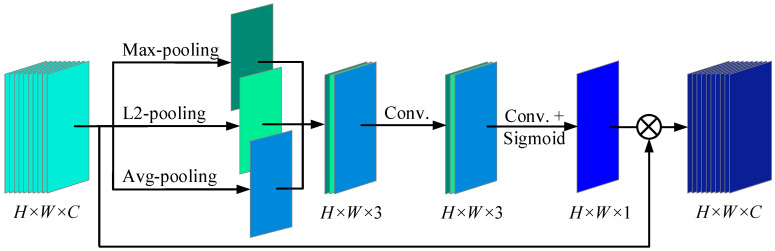
Depthwise convolution spatial attention module.

For spatial attention, GAP and GMP are commonly employed to identify important spatial positions and enhance the feature map accordingly. In the paper, L2-pooling is considered to dig local feature information, and max-pooling and avg-pooling are united to design a depthwise convolution spatial attention module as shown in Figure 3. Firstly, max-pooling, L2-pooling, and avg-pooling are applied along the channel axis to generate spatial feature maps. Since the operations are performed only along the channel axis, there is no need to define traditional 2D convolution kernels or stride parameters. Secondly, the above spatial feature maps are spliced in turn to form a feature map with a *H* × *W* × 3 feature map, and then convolve through two convolution layers with different kernel sizes. Finally, a sigmoid function is used to generate the spatial weight map. The depthwise convolution spatial attention module is computed as follows:

(10) $$X^{\prime \prime }=\mathrm{Sigmoid}(\mathrm{Conv}(\mathrm{Conv}([\max\_p(X^{\prime }),\;L2\_p(X^{\prime }),\mathrm{avg}\_p(X^{\prime })];\;k\times k\times 3,\;s);\;k^{\prime }\times k^{\prime }\times 1,\;s^{\prime }))\cdot X^{\prime }$$

where *X’* = {*X*_1_′, *X*_2_′, …, *X_c_*’, …}, respectively. *K* × *k* × 3 and *k*’ × *k*’ × 1 are the sizes of the convolution kernel in two convolution layers, and *s* and *s*’ are the corresponding strides, respectively.

### 3.2. Network Structure of Two-Stream 2DCNNs with DCBA Module

**Figure 4:**
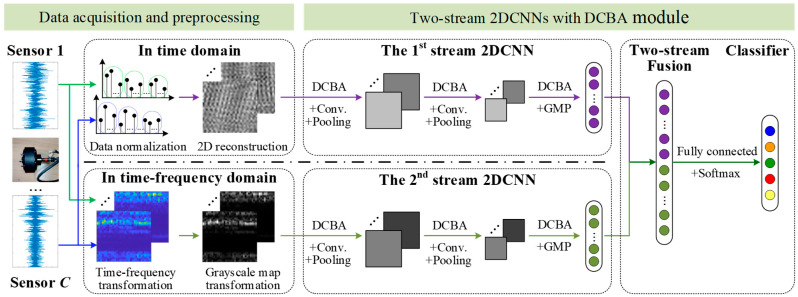
Network structure of two-stream 2DCNNs with DCBA module.

To accurately identify faults in each in-wheel motor of the four-wheel independent driving system under real-world conditions, a two-stream 2DCNN network with DCBA modules is proposed, and its architecture is illustrated in Figure 4. Each stream 2DCNN permits different input data: the first stream input data are the grayscale maps of time-domain signals reconstructed by two dimensions, and the second stream input data are the grayscale maps of the time–frequency transformation matrix, the signals of which must come from multiple sensors for monitoring the same in-wheel motor. The basic architecture of each stream in the 2DCNN consists of three DCBA modules, two convolutional layers, two pooling layers, and a flattening layer. Finally, the results of the two-stream 2DCNNs are fused by the fully connected layer, and then the different faults of the corresponding in-wheel motor are classified.

In the data acquisition and preprocessing stage, signals from multiple sensors monitoring the same in-wheel motor are categorized by fault severity and preprocessed in different domains. For the first-stream 2DCNN, the one-dimensional time signal of each sensor is normalized firstly between 0 and 255, and takes the integers through a round-function operation. Secondly, the characteristic frequencies of potential faults are analyzed, and the lowest frequency is selected to determine the corresponding number of sampling points. At least three times this number of points are then used to form a single sample. Let *H* be the number of sample measuring points; the measuring points of *H × W* are used to construct a two-dimensional array, where the row represents the measuring points of each sample, and the column represents the different samples. In general, the conversion is referred to as 2D reconstruction. Since the normalization range is aligned with the pixel intensity range of a grayscale image, the time-domain signal from a sensor can be transformed into an *H × W* grayscale image. If there are *C* sensors for monitoring the same in-wheel motor, a three-dimensional input map of *H* × *W* × *C* is structured as the input data of the first stream of the 2DCNN. For the second stream of the 2DCNN, the time-domain signal from a sensor is transformed into a time–frequency matrix using methods such as short-time Fourier transform (STFT), wavelet transform, Wigner–Ville distribution, Hilbert–Huang transform, or Gabor transform. To ensure the synchronization of the measuring points in the time and time–frequency domains, the same measuring points of *H* × *W* in the time domain must be used, but the number of rows and columns of the time–frequency transformation can be calculated according to the time resolution, frequency resolution, sampling frequency, and time–frequency transform method and its parameters. Then the resulting time–frequency matrix can be a *H* × *W* matrix or not. Next, the values representing the magnitude, energy, or power spectral density of the signal at specific time–frequency points are normalized to the range [0, 255], and the corresponding grayscale image is generated. Similarly, a three-dimensional input map is acquired as the input data of the second stream of the 2DCNN. Obviously, the input three-dimensional grayscale maps of two-stream 2DCNNs are different.

For two-stream 2DCNNs, the basic structure of each stream of the 2DCNN is the same. To guide subsequent convolutional operation for focusing on more fault features by weighting the input features, a DCBA module is inserted before each convolution layer. This is because the driving conditions are changeable and the operating environment is harsh, and there is serious noise in all monitoring signals of in-wheel motors. Then a DCBA module is first performed, and a convolutional block is operated. This process is referred to as a weighted convolution block. Each stream of the 2DCNN is configured with two weighted convolution blocks, and the corresponding kernels and strides can be set according to the specific task. Next, the third DCBA module is applied to guide the subsequent flattening process, enabling the network to focus on more salient features. The three-dimensional grayscale map from each stream of the 2DCNN is then flattened into a one-dimensional feature vector. Finally, the one-dimensional feature vectors from two-stream 2DCNNs are connected in series to form a comprehensive feature vector, which includes time-domain and time–frequency-domain features. The softmax function is employed to design a fault classifier.

### 3.3. Diagnosis Model of In-Wheel Motors’ Faults

Considering the real working condition of a four-wheel independent driving vehicle, vibrations from two sensors are studied to diagnose the mechanical faults of in-wheel motor. Moreover, the fault features and frequencies of in-wheel motor in the time domain and time–frequency domain are analyzed, 1024 of the measuring points are regarded as an observation unit, and a three-dimensional grayscale map with 32 × 32 × 2 dimensions in the time domain is firstly structured as the input data of the first stream of the 2DCNN. In the time–frequency domain, STFT is selected to form a three-dimensional grayscale map of 33 × 33 × 2 dimensions as the input data of the second stream of the 2DCNN, where the window type is a Hanning window, the window length is 64, and the frame shift is 32.

For more weighted feature extraction of each in-wheel motor in a four-wheel independent driving system, six DCBA modules are integrated into two-stream 2DCNNs. To simplify the calculation, each DCBA module has the same role, and the parameters are set as the same, as shown in Table 1.

To enable intelligent identification of various fault types, a diagnosis model for each in-wheel motor is constructed, and the detailed configurations of the two-stream 2DCNNs with DCBA modules are presented in Table 2. Moreover, a softmax function is applied in the final layer to compute the probability distribution over all fault categories.

## 4. Performance Verification

To verify the effectiveness of the proposed two-stream 2DCNNs with DCBA module for diagnosing a fault of each in-wheel motor, two case of in-wheel motor monitoring data from the different self-made experiment benches have been studied, including Case 1: the faults data of in-wheel motor under static interference and Case 2: the faults data of in-wheel motor under dynamic disturbance.

### 4.1. Case 1: In-Wheel Motor Experiment Under Static Interference

**Figure 5:**
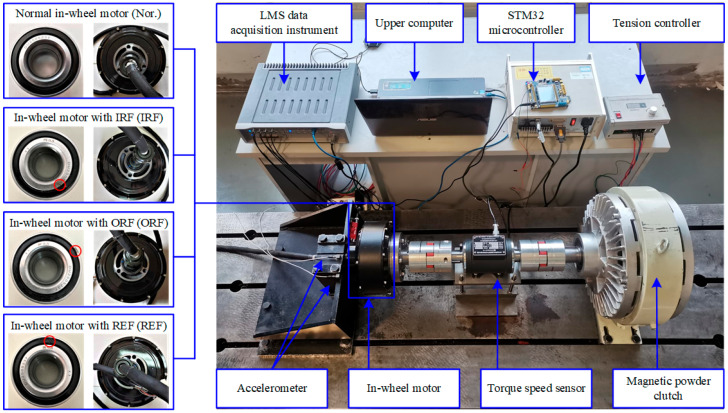
Case 1: experimental bench of in-wheel motor under static interference.

Figure 5 shows the experimental bench of an in-wheel motor under static interference that is mainly composed of an in-wheel motor, an inverter, and a magnetic powder brake. A set of power batteries provides the electricity power, a STM microcontroller is linked with the inverter to control the rotating speed, and a tension controller is used to exert a load on the magnetic powder brake. Moreover, two accelerometers (type: PCB333B30; sensitivity: 100 mV/g) are mounted on the horizontal and vertical directions of the bracket that holds the stator axis of an in-wheel motor, and a torque speed sensor is jointed to measure the real torque and speed. To describe the fault states of an in-wheel motor quantitatively, three bearings (type: DU2505237) dedicated in the in-wheel motor are artificially processed with a single point of damage (width 0.3 mm and depth 0.15 mm) across the inner ring, rolling element, and outer ring in sequence, then are fixed tightly on the stator axis of each in-wheel motor by professionals. In the process of the experiment, a normal in-wheel motor (Nor.) and three in-wheel motors with an inner race fault (IRF), outer race fault (ORF), and rolling element fault (REF) were operated at the working speeds of 100, 200, …, 700 r/min (or thereabouts) under the working loads of 0, 10, 20, and 30 N·m. The experiment data were collected using an LMS data acquisition instrument with a sampling frequency of 12.8 kHz and sampling time of 20 s. In this experiment, a 16-channel LMS multi-functional data acquisition system was employed, connected to the accelerometers mounted on the test rig, to synchronously capture the vibration signals through the data acquisition terminal. Considering the intermittent strong interference experienced by the in-wheel motor during operation, piezoelectric accelerometers from Kistler were selected for radial vibration signal detection. The sensors were mounted in the vertical and horizontal directions on the base near the bearing mounting positions of the in-wheel motor, enabling the acquisition of multi-source vibration signal data.

To evaluate the diagnosability, stability, generalization, and superiority of the proposed diagnosis model, holdout-validation, cross-validation and ablation experiments were used successively.

#### 4.1.1. Holdout-Validation of Diagnosis Model Under the Same Working Condition of Case 1

**Figure 6:**
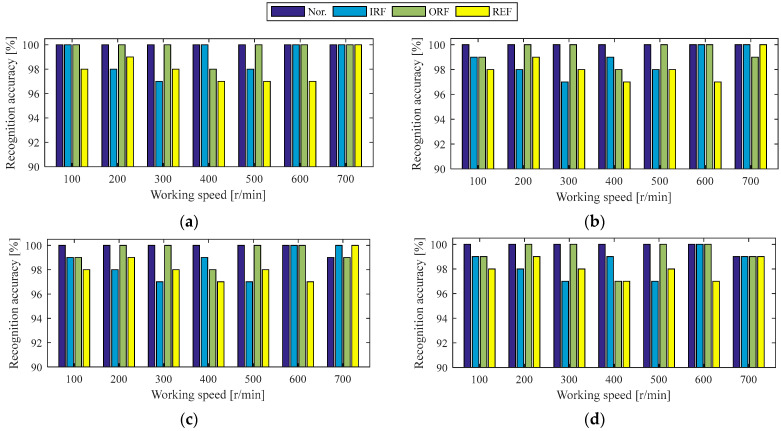
Recognition accuracies of four states of in-wheel motor under the same working conditions of the Case 1 experiment: (**a**) 0 N·m load, (**b**) 10 N·m load, (**c**) 20 N·m load, (**d**) 30 N·m load.

Since the Case 1 experiment involved seven speeds and four roads, 28 working conditions were assessed in total, and holdout-validation was used to verify the diagnosis model under the same working conditions. For each working condition, the experimental data were processed into 250 observation samples using the set-out method, and these samples were randomly divided in a 6:4 ratio into training and testing sets. The next step in the workflow was to train the diagnostic model for each working condition. Firstly, the hyperparameters of 2DCNN were set, the batch size was set to 64, the number of epochs was set to 100, the optimization algorithm was the Adam optimizer, and the learning rate was 1 × 10^−4^. Additionally, the implementation was conducted in Python 3.7, and the deep learning framework used was TensorFlow 2.7.0. Secondly, the normal in-wheel motor and three faulty in-wheel motors under the same working conditions were regarded as four states of the in-wheel motor, and the training samples of the four states under each working condition were packed together. For example, under the working condition of 100 r/min rotational speed and 10 N·m load, there are four states of in-wheel motors. With 150 training samples per state, this results in a total of 600 training samples per working condition. Similarly, 400 testing samples under each working condition can be obtained. When the training samples for each working condition are fed into the proposed two-stream 2DCNNs, the corresponding diagnostic model is trained. Finally, the testing samples under the same working condition were used to test the recognition accuracy of each state of the in-wheel motor, as shown in Figure 6.

Clearly, the diagnostic model incorporating the two-stream 2DCNNs and DCBA module achieves a remarkable recognition accuracy exceeding 97% for each state of the in-wheel motor under identical working conditions, particularly for the normal state, which is accurately identified except at a working speed of 700 r/min under loads of 20 and 30 N·m. On average, the recognition accuracies for the four states exceed 98%. From the working load point of view, the average diagnosis accuracy under 0 N·m load is higher. However, the average diagnosis accuracy decreases slightly with the increase in working load, but it can be maintained at 98.89%. From the working speed point of view, the average diagnosis accuracies at the working speeds of 100, 200, 600, and 700 r/min always reach above 99%. By contrast, the average diagnosis accuracy at the working speed of 400 r/min is lower. Analysis of the vibration signals at various working speeds confirms that the fault frequency of the in-wheel motor at 400 r/min closely matches the natural frequency of the experimental setup, leading to significant noise interference. Consequently, the recognition accuracies for the four states at working speeds of 300, 400, and 500 r/min exhibit noticeable variations; however, these variations remain within acceptable engineering limits.

#### 4.1.2. Cross-Validation of Diagnosis Model Under Similar Working Conditions to the Case 1 Experiment

In practical applications, the working condition of each in-wheel motor is varied, then cross-validation is used to verify the stability and generalization of the proposed diagnosis model under similar working condition. Here, the so-called similar working condition is that the working speed is close or the working load is close. Accordingly, two types of cross-validation schemes are designed. In the first scheme, the working load is fixed, and experimental data from two non-consecutive speeds are used as training samples, while the data from the intermediate speed are used for testing. For example, the experimental data at the speeds of 100 r/min and 300 r/min under 0 N·m load are regarded as training data, labeled as 100 and 300, and the experimental data at the speeds of 200 r/min under 0 N·m load are regarded as testing data. In the second scheme, the working speed is held constant, and data from two non-consecutive load levels are used for training, while the data from the intermediate load are reserved for testing. For example, the experimental data at the speed of 100 r/min under 0 N·m and 20 N·m load are regarded as training data, labeled as 0 and 20, and the experimental data at the speeds of 100 r/min under 10 N·m load are regarded as testing data. The above 2DCNN hyperparameters, optimization algorithm, learning rate, and framework remain unchanged during the implementation of both cross-validation schemes. Table 3 shows the concrete schemes and results of cross-validation of the diagnosis model.

On the whole, the diagnostic results of the two cross-validation schemes are satisfactory; the recognition accuracy of each diagnosis model is more than 92.8% for each state of the in-wheel motor under similar working conditions, especially for the normal state of in-wheel motor, which is basically identified with an accuracy of above 95%. Certainly, compared with the holdout-validation results obtained under identical working conditions, the cross-validation accuracies are slightly lower; however, they still meet the required engineering standards. On the average, the diagnosis model under similar working conditions basically has a recognition accuracy of about 95%, but there is only one condition where the average accuracy is lower than 94%, at the speeds of 300 and 500 r/min under a 20 N·m load, which was used to train the diagnosis model. An integrated analysis suggests that the proximity of the resonance frequency at a rotational speed of 400 r/min significantly affects the experimental data. Moreover, the recognition accuracies of the two cross-validation schemes are different. For the diagnosis results of the first scheme, 25% of in-wheel motor states under similar working conditions were correctly recognized with an accuracy over 96%, with the highest reaching 98%. At the same time, the recognition accuracy of less than 94% was also seen more. In the second scheme, the highest recognition accuracy reaches 97.2%, and the average accuracy across all models remains relatively high. It follows then that the diagnosis model of the two-stream 2DCNNs with a DCBA module is more sensitive to the working speed than the working load.

#### 4.1.3. Ablation Experiment Under the Same Working Conditions as the Case 1 Experiment

**Figure 7:**
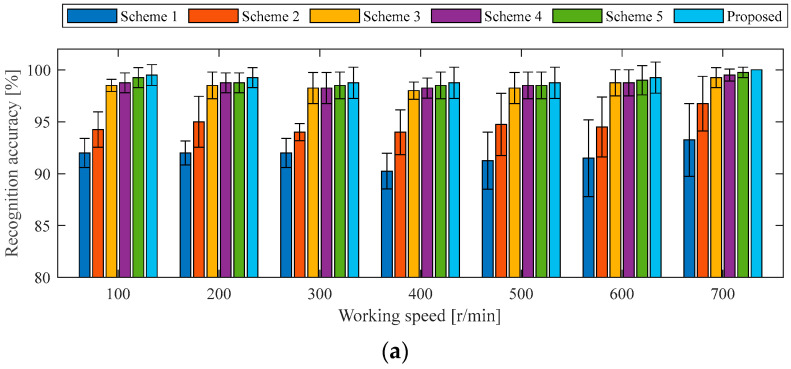

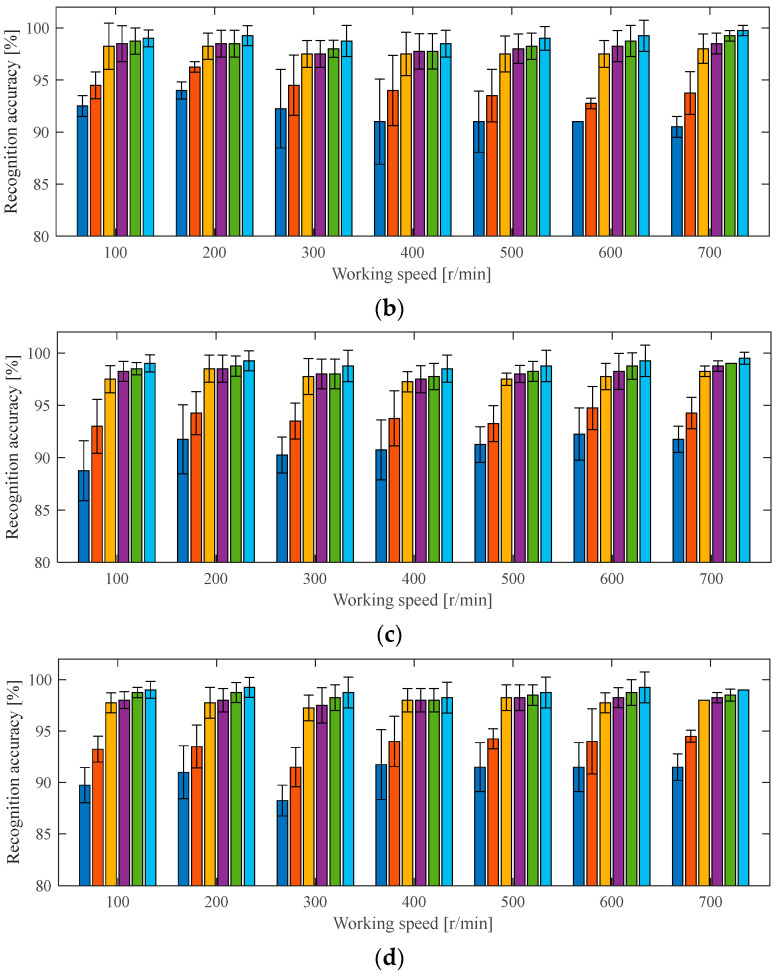
Average accuracies of various schemes for recognizing four states of the in-wheel motor under the same working conditions as the Case 1 experiment: (**a**) 0 N·m load, (**b**) 10 N·m load, (**c**) 20 N·m load, (**d**) 30 N·m load.

To further validate the effectiveness of the two-stream 2DCNN architecture with the DCBA module, ablation experiments were conducted by removing or replacing specific components of the proposed model. Five different experimental schemes were designed for comparative analysis. In reference to the original model, each of the two-stream 2DCNN branches is individually removed to evaluate the performance of a single-stream 2DCNN with the DCBA module. Scheme 1 evaluates the model using only the first 2DCNN stream, and Scheme 2 evaluates the model using only the second 2DCNN stream. Moreover, the DCBA module of two-stream 2DCNNs is replaced by SENet, ECANet, and CBAM in turn, and the corresponding models are denoted as Scheme 3, Scheme 4, and Scheme 5. In all of the above schemes, the 2DCNN-related parameters are kept consistent with those of the original model, while certain architecture-specific settings are adjusted independently. For example, the convolution kernel’s size of ECANet in Scheme 4 is set to 3, and the convolution kernel’s size and the stride of CBAM in Scheme 5 are assigned as 3 and 1. The same training samples of holdout-validation are used to train each design scheme, and the testing samples of holdout-validation under the same working conditions are used to verify the recognition accuracy of each state of the in-wheel motor. To facilitate the comparison of overall performance across different schemes, the recognition accuracies of the four in-wheel motor states are averaged, and the dispersion across each working condition is evaluated, as illustrated in Figure 7.

Regarding the average accuracies and dispersion degrees for recognizing four states of an in-wheel motor under identical working conditions, the proposed two-stream 2DCNNs with the DCBA module achieve the highest performance, followed by two-stream 2DCNNs incorporating CBAM, ECANet, and SENet modules. The single-stream 2DCNN with the DCBA module exhibits inferior results. Specifically, the recognition accuracy of each diagnosis model established by the proposed method is more than 98.25%, and the maximum dispersion degree is less than 1.5%. For Scheme 3, Scheme 4, and Scheme 5, the network structure of two-stream 2DCNNs is inherited, the different AMs perform state classifications with accuracies of no less than 97.25%, but the maximum dispersion degree is expanded to 2.22%. Therefore, the proposed DCBA module organically combines classical AM with modern image processing techniques in the process of fault feature extraction, and adds L2-pooling to enhance the extraction ability of local fault feature in changeable driving conditions and a harsh operating environment. As far as Scheme 1 and Scheme 2 are concerned, single-domain signals are used to build the diagnosis model under the same working conditions, the recognition accuracies drop to around 90%, and the maximum dispersion degree is increased to 4.08%. This result demonstrates that two-stream 2DCNNs are complementary.

### 4.2. Case 2: In-Wheel Motor Experiment Under Dynamic Disturbance

**Figure 8:**
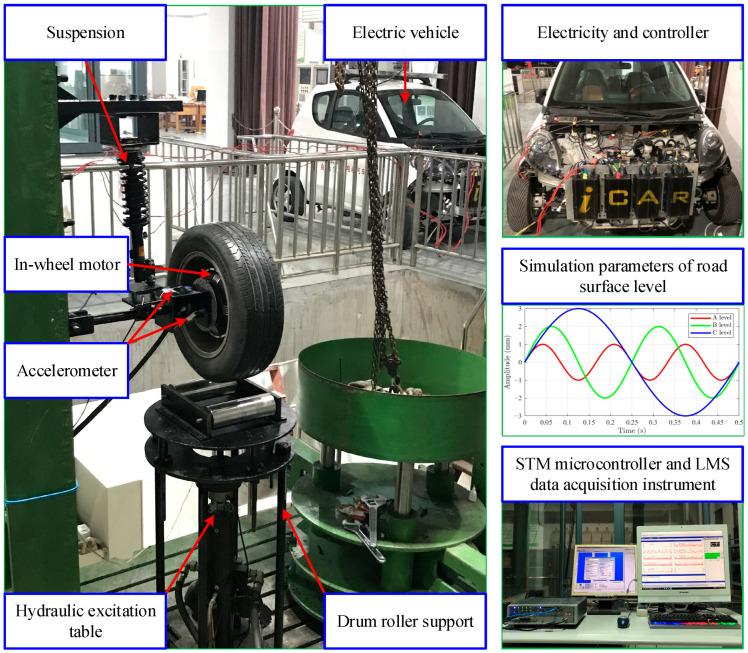
Case 2 experimental bench of in-wheel motor under dynamic disturbance.

Figure 8 shows the experimental bench for testing in-wheel motors under dynamic disturbances, designed to closely simulate actual operating scenarios. First, the stator of the in-wheel motor is mounted on the suspension using a vehicle-mimetic connection method, while the motor is embedded in the wheel hub with its rotor fixed to the hub. An electric vehicle is used to supply the electricity and controller. Second, the hydraulic excitation table is controlled to vibrate at a certain amplitude and frequency, such as 1 mm and 6 Hz, 2 mm and 4 Hz, or 3 mm and 2 Hz, for simulating the road surface levels A, B, and C, respectively. The table’s initial height is adjusted via pressure sensor readings between the suspension and fixture to achieve a vertical load of 0.5 T. The STM microcontroller is employed to replace the accelerator pedal for repeating the same working speed. Additionally, a drum roller support is installed, contacting the rotating tire at the top and fixed to the hydraulic table at the bottom. Finally, the same in-wheel motors used in the experiment of Case 1 are orderly exchanged to run in the approximate operating scenario, including the working speeds of 100, 200, …, 700 r/min, the road surfaces of A, B and C level and the vertical load of 0.5 T. Two accelerometers are used to collect the vibration signals in the horizontal and vertical directions of the bracket that holds the stator axis of the in-wheel motor. The sampling frequency is 12.8 kHz and the sampling time is 20 s.

Similarly, the proposed diagnosis model is evaluated under dynamic disturbances using holdout-validation, cross-validation, and a series of ablation experiments.

#### 4.2.1. Holdout-Validation of Diagnosis Model Under the Same Working Conditions as the Case 2 Experiment

**Figure 9:**
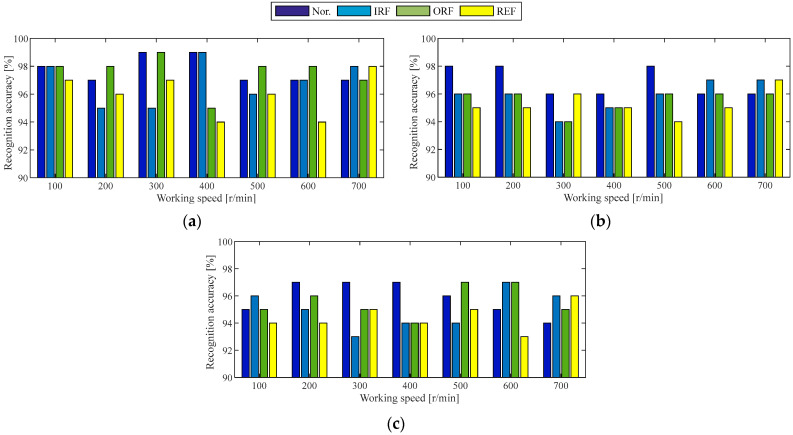
Recognition accuracies of four states of in-wheel motor under the same working conditions as the Case 2 experiment: (**a**) A-level road surface, (**b**) B-level road surface, (**c**) C-level road surface.

The experimental setup in Case 2 comprises seven rotational speeds and three road surface types, resulting in a total of 21 distinct operating conditions. The preprocess method of each observation sample, allocation proportion of training and testing samples, hyperparameters of 2DCNN, algorithm language, and deep learning framework remain the same as the experimental verification of Case 1. For each operating condition, a two-stream 2DCNN model integrated with the DCBA module is trained on 600 samples per motor state. The remaining 400 samples per condition are allocated for testing, with state-specific recognition accuracy illustrated in Figure 9. The remaining 400 samples under the corresponding working conditions are used to test the recognition accuracy of each state, as shown in Figure 9.

The proposed diagnostic model achieves an accuracy exceeding 93% for each in-wheel motor state under identical operating conditions. On average, the recognition accuracy of each state of the in-wheel motor is over 96%. From the working speed point of view, the average diagnosis accuracy of the four states of the in-wheel motor at each working speed is exactly 96%. From the road surface point of view, the average diagnosis accuracy under the A-level road surface is the highest, exceeding 97%. Next, the average diagnosis accuracy under the B-level road surface is close to 96%. By contrast, the average diagnosis accuracy under the C-level road surface is lower, but still remains above 95%. Through analyzing the original vibration signals under the road surface at the A, B, or C level, it is confirmed that the dynamic disturbance under the C-level road surface is large, and the fault features of the in-wheel motor are severely overwhelmed.

#### 4.2.2. Cross-Validation of Diagnosis Model Under Similar Working Conditions as the Case 2 Experiment

Modeled after the cross-validation schemes of the Case 1 experiment, two categories of cross-validation schemes are designed to verify the performance of the proposed diagnosis model under similar working conditions of dynamic disturbance. The first scheme is that the road surface of the in-wheel motor is fixed; the experimental data at two alternate speeds are processed into training samples; and the experimental data at the speed between them are regarded as testing samples. The second scheme is that the working speed is fixed, the experimental data under two road surfaces are processed into training samples, and the experimental data under other road surfaces between them are regarded as testing samples. Both models were trained and tested accordingly, with the comparative diagnostic performance metrics detailed in Table 4.

The average accuracies of two categories of cross-validation from the Case 2 experiment are 94.0% and 93.7%, and the recognition accuracy of each diagnosis model is more than 91.6% for each state of the in-wheel motor. Compared with the holdout-validation from the same experiment in Case 2, the recognition accuracies of most cross-validation models show a decrease, but the average accuracy has only dropped by 2.16%. Compared with cross-validation from the Case 1 experiment, the average accuracy of cross-validation from the Case 2 experiment has decreased by 1.22%. Because cross-validation incorporates diverse road surfaces, fault features become concealed by dynamic disturbances in the Case 2 experiment. Consequently, although diagnostic accuracies show slight declines, all results still satisfy engineering requirements.

#### 4.2.3. Ablation Experiment Under the Same Working Conditions as the Case 2 Experiment

**Figure 10:**
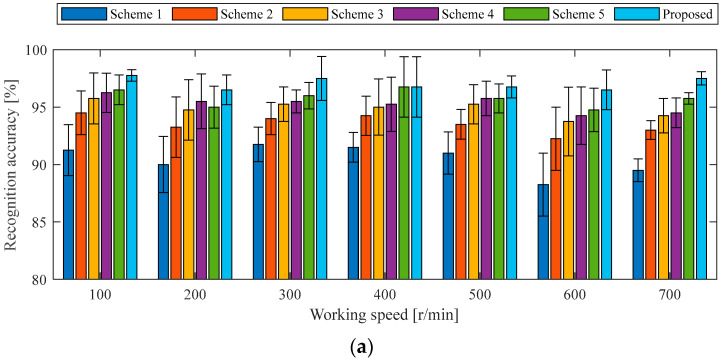

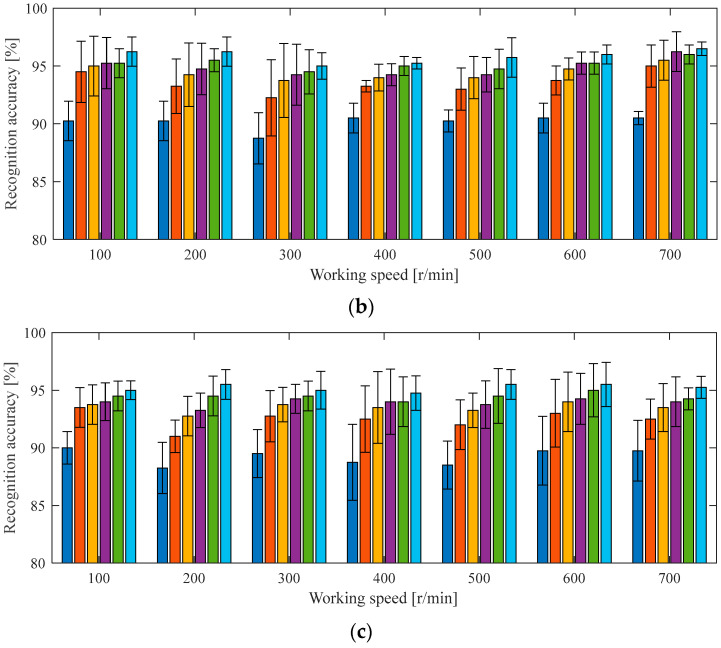
Average accuracies of various schemes for recognizing four states of the in-wheel motor under the same working conditions as the Case 2 experiment: (**a**) A-level road surface, (**b**) B-level road surface, (**c**) C-level road surface.

Five ablation schemes from Case 1 were implemented. Using holdout-validation data from Case 2 under identical operating conditions, training samples were applied to each scheme, while testing samples evaluated diagnostic accuracy across all road surfaces and speed ranges. Figure 10 shows the average accuracies and dispersion degrees for recognizing four states of the in-wheel motor under the same working conditions as the Case 2 experiment using five ablation schemes and the proposed method.

The proposed method demonstrates accuracy rates between 94.75% and 97.75%; the mean accuracy achieved was 96.04%. For the five ablation schemes, the average accuracies from Scheme 1 and Scheme 5 dropped to the average levels of 89.94%, 93.19%, 94.27%, 94.70%, and 95.14%, respectively. As such, the performance of the two-stream 2DCNNs with a DCBA module is outstanding, followed by those with CBAM, ECANet, and SENet modules. In contrast, a single-stream 2DCNN with a DCBA module shows significantly poorer performance, especially the first stream of the 2DCNN with time-domain signals. In terms of dynamic disturbance, the average accuracies used by the proposed method under A-, B-, and C-level road surfaces achieve the average levels of 97.04%, 95.86%, and 95.21%, respectively. As the road surface interference intensifies, the diagnostic accuracy decreases. As seen in Figure 10, it is easy to observe similar trends for the five ablation schemes. This indicates that the proposed DCBA module is still better than the CBAM, ECANet, and SENet modules for extracting the in-wheel motors’ features under dynamic disturbance, and the proposed two-stream 2DCNNs effectively integrate high-quality resources from the time domain and time–frequency domain.

### 4.3. Discussion

The superiority of the proposed fault diagnosis method stems from the DCBA module’s ability to extract hierarchical features—including global salient features, localized micropatterns, and domain-average characteristics—from vibration-reconstructed grayscale images across multiple transform domains. For the monitoring data of the in-wheel motor under static interference and dynamic disturbance, the DCBA module is used to improve the average accuracies of 0.9%, 1.34%, and 1.77%, respectively, compared to the CBAM, ECANet, and SENet modules, and as such it can be said that the DCBA module accurately captures the nature features of bearing faults, and is suited for feature extraction in changeable driving conditions and harsh operating environments. Moreover, the novel network structure of two-stream 2DCNNs learns high-quality feature representations from the time domain and time–frequency domain through multiple layers of DCBA, convolution, pooling, and other operations, and integrates the information through a fully connected layer, then achieves complementary advantages and increases the average accuracy to over 96%. Compared with the network structure of a single-stream 2DCNN with a DCBA module, the average recognition accuracy has increased by at least 2.85%. Facing the highly competitive EV market, the in-wheel motor fault diagnosis method proposed in this paper has significant theoretical guidance and practical significance for enhancing vehicle operation safety.

To demonstrate the superiority of the proposed method, four states of in-wheel motor are recognized by the above two cases for a comparison. The external interference of the experiment expanded from static to dynamic, the working speed increased from slow to fast, and the working load rose from no-load to full-load, comprehensively simulating the actual operating environment of an in-wheel motor in EVs. Moreover, three experimental verification methods, holdout-validation, cross-validation and ablation experiments, were performed to evaluate the diagnosability, stability, generalization, and superiority of the proposed diagnosis model. Figure 6 and Figure 9 demonstrate that the holdout-validation of the diagnosis model achieves an average accuracy of more than 96% under the same working conditions, and Table 3 and Table 4 show the cross-validation of the diagnosis model realizes an average accuracy of more than 93% under similar working condition. This shows good evidence that the proposed diagnosis model has high diagnosability, robust stability, and strong generalization. Figure 7 and Figure 10 demonstrate that the proposed diagnosis method is superior to all schemes in the ablation experiment.

## 5. Conclusions

This paper proposes a novel fault diagnosis method based on two-stream 2DCNNs with a depthwise convolution block attention (DCBA) module, aiming to accurately identify faults in in-wheel motors within four-wheel independent driving systems under variable driving conditions and harsh operating environments. The correctness and applicability of the proposed method are demonstrated through comprehensive experimental studies.

The method exhibits two key advantages. First, the DCBA module effectively enhances feature expression and model robustness by extracting multi-scale features—such as prominent, local, and average information—from grayscale images reconstructed from vibration signals. This enables the model to better adapt to variations in working conditions and significantly improves diagnostic accuracy across different tasks. Second, the two-stream 2DCNN architecture integrates complementary information from both time-domain and time–frequency domain signals, enabling more comprehensive fault pattern recognition. Moreover, this structure can be easily extended to multi-stream frameworks for incorporating features from additional domains, offering strong scalability.

The effectiveness of the proposed method has been validated through two practical experimental cases involving four motor states, seven working speeds, four load levels, and three road surface types. The results demonstrate high recognition accuracy, low dispersion, and strong robustness under complex conditions. Comparative experiments with models based on SENet, ECANet, and CBAM further confirm its superior performance.

These findings highlight the potential of the proposed method for real-world applications in electric vehicles equipped with in-wheel motors. Future work will focus on implementing the method in actual vehicle systems and exploring its performance in dynamic, real-time driving scenarios.

## Figures and Tables

**Table 1:** Parameters of DCBA modules.

Module	Layer Type	Kernel Size	Stride
Channel attention	max-pooling	2 × 2	2
	L2-pooling	2 × 2	2
	avg-pooling	2 × 2	2
	Conv	1 × 1 × 3	1
Spatial attention	max-pooling	/	/
	L2-pooling	/	/
	avg-pooling	/	/
	Conv1	3 × 3 × 3	1

**Table 2:** Detailed setting of two-stream 2DCNNs with DCBA module.

Process	Layer Type	Kernel Number @ Size/Stride/Padding	Output Size
1st-stream 2DCNN	Input		2 @ 32 × 32
	DCBA module		2 @ 32 × 32
	Conv1	16 @ 3 × 3/1/1	16 @ 32 × 32
	Pooling1	2 × 2/2/0	16 @ 16 × 16
	DCBA module		16 @ 16 × 16
	Conv2	32 @ 3 × 3/1/1	32 @ 16 × 16
	Pooling2	2 × 2/2/0	32 @ 8 × 8
	DCBA module		32 @ 8 × 8
	GMP		32 @ 1 × 1
2nd-stream 2DCNN	Input		2 @ 33 × 33
	DCBA module		2 @ 33 × 33
	Conv1	16 @ 3 × 3/1/1	16 @ 33 × 33
	Pooling1	2 × 2/2/0	16 @ 16 × 16
	DCBA module		16 @ 16 × 16
	Conv2	32 @ 3 × 3/1/1	32 @ 16 × 16
	Pooling2	2 × 2/2/0	32 @ 8 × 8
	DCBA module		32 @ 8 × 8
	GMP		32 @ 1 × 1
Fusion and decision	Two-stream fusion		64 @ 1 × 1
	Fully connected layer		5 @ 1 × 1

**Table 3:** Cross-validation schemes and results of the diagnosis model under similar working conditions to the Case 1 experiment.

Condition of Training Data		Condition of Testing Data		Recognition Accuracy of Each State				Average Accuracy
Load	Speed	Load	Speed	Nor.	IRF	ORF	REF	
0	100 and 300	0	200	98.0%	94.8%	96.8%	96.0%	96.4%
0	200 and 400	0	300	96.4%	94.0%	95.2%	94.0%	94.9%
10	200 and 400	10	300	97.6%	94.4%	96.0%	95.2%	95.8%
10	300 and 500	10	400	95.6%	94.0%	94.8%	94.0%	94.6%
20	300 and 500	20	400	94.4%	93.2%	94.0%	93.6%	93.8%
20	400 and 600	20	500	95.2%	93.2%	94.4%	94.4%	94.3%
30	400 and 600	30	500	95.6%	93.6%	96.0%	94.8%	95.0%
30	500 and 700	30	600	96.0%	94.0%	95.6%	95.2%	95.2%
0 and 20	100	10	100	96.8%	94.4%	96.0%	96.8%	96.0%
0 and 20	200	10	200	96.4%	92.8%	95.6%	93.6%	94.6%
0 and 20	300	10	300	97.2%	93.2%	95.2%	95.2%	95.2%
10 and 30	400	20	400	96.4%	93.6%	95.6%	94.4%	95.0%
10 and 30	500	20	500	96.0%	94.8%	95.6%	95.2%	95.4%
10 and 30	600	20	600	95.6%	94.4%	95.2%	94.0%	94.8%
10 and 30	700	20	700	95.6%	94.4%	96.8%	94.4%	95.3%

**Table 4:** Cross-validation schemes and results of diagnosis model under similar working conditions as the Case 2 experiment.

Condition of Training Data		Condition of Testing Data		Recognition Accuracy of Each State				Average Accuracy
Load Surface	Speed	Load Surface	Speed	Nor.	IRF	ORF	REF	
A	100 and 300	A	200	95.6%	92.4%	95.6%	95.2%	94.7%
A	200 and 400	A	300	96.0%	93.2%	94.8%	94.0%	94.5%
A	300 and 500	A	400	96.4%	92.8%	94.4%	94.4%	94.5%
B	200 and 400	B	300	95.6%	93.2%	94.8%	94.8%	94.6%
B	300 and 500	B	400	95.2%	92.8%	94.0%	93.6%	93.9%
B	400 and 600	B	500	94.0%	92.4%	93.6%	93.6%	93.4%
C	300 and 500	C	400	94.0%	91.6%	93.6%	93.2%	93.1%
C	400 and 600	C	500	94.4%	92.4%	94.4%	94.4%	93.9%
C	500 and 700	C	600	93.6%	92.8%	94.0%	94.0%	93.6%
A and C	100	B	100	95.2%	92.8%	94.8%	95.2%	94.5%
A and C	200	B	200	94.8%	92.8%	95.2%	94.0%	94.2%
A and C	300	B	300	95.6%	92.0%	94.4%	94.4%	94.1%
B and C	300	A	300	94.0%	93.2%	93.6%	92.8%	93.4%
B and C	400	A	400	94.4%	92.8%	94.0%	92.4%	93.4%
B and C	500	A	500	94.0%	94.0%	92.8%	91.6%	93.1%
A and B	500	C	500	94.0%	94.4%	93.2%	93.2%	93.7%
A and B	600	C	600	94.4%	93.2%	92.4%	92.8%	93.2%
A and B	700	C	700	94.8%	93.2%	93.6%	94.0%	93.9%

## Data Availability

The original contributions presented in this study are included in the article. Further inquiries can be directed to the corresponding author.
